# Diagnostic Yield of Malignant Pleural Effusion in Various Primary and Metastatic Cancers: Insights Across Cancer Subtypes

**DOI:** 10.1155/pm/8469617

**Published:** 2026-05-13

**Authors:** Santosh Basyal, Antony D. Rawindraraj, Rahul Parajuli, Shardul Bhattarai, Laxman Wagle, Kritick Bhandari, Vikas Pathak

**Affiliations:** ^1^ Department of Internal Medicine, Nepal Medical College and Teaching Hospital, Kathmandu, Nepal; ^2^ Department of Internal Medicine, University of Missouri, Columbia, Missouri, USA, missouri.edu; ^3^ Department of Pulmonology and Critical Care, Hospital for Advanced Medicine and Surgery (HAMS), Kathmandu, Nepal; ^4^ Department of Internal Medicine, Ascension Saint Agnes Hospital, Baltimore, Maryland, USA; ^5^ Department of Internal Medicine, Kist Medical College Teaching Hospital, Lalitpur, Nepal; ^6^ Interventional Pulmonology and Critical Care Medicine, Virginia Institute of Lung Diseases, Mechanicsville, Virginia, USA

**Keywords:** cytology, malignant pleural effusion, medical thoracoscopy, thoracoscopy, video-assisted thoracoscopic surgery (VATS)

## Abstract

**Objective/Background:**

Pleural fluid cytology remains a cornerstone in diagnosing malignant pleural effusions (MPEs); however, its diagnostic yield varies considerably across cancer subtypes. This study is aimed at evaluating the diagnostic performance of cytology stratified by malignancy type.

**Methods:**

We conducted a retrospective study at a tertiary referral center in Raleigh, North Carolina, enrolling patients aged 18–100 years who underwent thoracentesis for suspected MPE between January 2015 and August 2018. MPE was defined by positive pleural fluid cytology, VATS findings, or histopathological confirmation. Variables analyzed included demographics, cytology results, thoracoscopic findings, and pleural biopsy histopathology.

**Results:**

Of the 779 pleural fluid samples, we included 667 patients (99 malignant and 482 benign); the remaining 86 samples included therapeutic thoracenteses, dry taps, and inadequate volume for cytology. Cytology diagnosed 86 cases of MPE (86.86%), while VATS identified additional eight cases. Overall cytology specificity was 99.8% (95% CI: 99.3%–100%). Adenocarcinoma demonstrated the highest cytologic sensitivity (90.24%), particularly in breast cancer–related MPE (67.44%). Among 482 cytology‐negative cases, 130 had documented cancer histories, most frequently lung cancer (17.69%), hematological malignancies (12.30%), and breast cancer (10.76%). Skin and prostate cancers were more likely to yield false‐negative cytology results. Malignant mesothelioma requires repeat cytology or VATS for definitive diagnosis in most cases.

**Conclusion:**

Pleural fluid cytology is highly specific and most sensitive for lung and breast adenocarcinomas. Combined cytology and VATS demonstrate superior diagnostic accuracy and should be the standard approach when initial cytology is negative and malignancy remains clinically suspected.

## 1. Introduction

Malignant pleural effusions (MPEs) are one of the strongest indicators of poor prognosis in patients diagnosed with cancer, with an average survival of 3–12 months depending on the primary tumor type [1]. Over 75% of MPEs are caused by malignancies involving the breast, lung, gastrointestinal tract, ovary, lymphomas, and blood. Bedside thoracentesis with analysis of pleural fluid via cytology is the most common initial investigation in patients with suspected MPEs. Compared with more advanced techniques, such as thoracoscopy, thoracentesis remains a simple, minimally invasive, cost‐effective procedure with low complication rates. Pleural fluid cytology plays a vital role in establishing a cause for the pleural effusion; however, the sensitivity and specificity of this diagnostic approach vary considerably. The reported diagnostic yield of pleural fluid cytology has a wide range, from 40% to 87%, and varies from institution to institution [2]. Classically, a lower diagnostic yield of pleural fluid cytology is associated with a low volume of fluid submissions, delayed analysis of pleural fluid after collection, and a lower extent of pleural involvement. However, improved diagnostic yields of pleural fluid cytology have been observed with repeated thoracentesis, increased volume of pleural fluid aspirations, and the combination of cytology results with percutaneous pleural biopsy results.

The primary goal of this study is to assess the diagnostic yield of pleural fluid cytology in patients with unilateral or bilateral pleural effusions between 2015 and 2018. Loveland et al. [3] analyzed 153 patients over a 12‐month period in Melbourne and Australia, which reported a high diagnostic yield with 67.2% sensitivity and 85.9% specificity for diagnosing malignant effusions using pleural fluid cytology. Our study analyzed a larger subset of patients, spanning a 3‐year period, who underwent thoracentesis and subsequent analysis of pleural fluid cytology to obtain the diagnostic yield of pleural fluid cytology. In addition, the study also attempts to differentiate the diagnostic yield of pleural fluid cytology based on the type of cancer resulting in MPE. This helps evaluate the role of different tumor histopathology in influencing the diagnostic yield of pleural fluid cytology. Recent technological advances, including visible‐light hyperspectral imaging and principal component analysis applied to cytological specimens, have begun to address the long‐standing limitations of morphology‐based pleural fluid analysis and may pave the way to more reproducible and sensitive diagnosis of MPEs [4].

Loveland et al. [3] reported that the accuracy of MPE diagnosis was higher in patients with lung adenocarcinoma (85.7% sensitivity), whereas the accuracy of diagnosis was lower for mesothelioma (45.5% sensitivity). This study differentiated the diagnostic yield of pleural fluid cytology based on different cancer subtypes, including adenocarcinoma, squamous cell carcinoma, small cell and nonsmall cell carcinomas, mesothelioma, ovarian carcinoma, lymphoma, breast carcinoma, and other malignancies.

To the best of our knowledge, the majority of the recent studies focused on obtaining the diagnostic yield of pleural fluid cytology were from centers around the world, including Australia, India, Pakistan, Nepal, Singapore, and Italy. Given the differences in patient populations between these countries and the United States, the present study offers a diagnostic yield that more accurately reflects the patient population treated within the US healthcare system.

One frequent consequence of advanced cancer is MPE. Increased fluid production and/or decreased fluid resorption can lead to pleural effusions. During the course of their disease, pleural effusion will occur in 15% of individuals with cancer due to malignant infiltration of the pleura [5]. In the United States, MPE is responsible for around 125,000 admissions annually, with inpatient fees alone costing almost $5 billion. It typically signals a poor prognosis because it represents an advanced stage or metastatic disease. Patients who present with MPE typically have a life expectancy of 3–12 months, depending on their comorbidities and the type of underlying malignancy [6]. Thoracentesis is the most common method for diagnosing MPE (60% on the first attempt); however, repeat thoracentesis, computed tomography–guided biopsy, or even thoracoscopy may be required [7]. Thoracoscopy is the preferred method in patients with suspected MPE who have negative cytology [8]. Lung cancer, breast cancer, and lymphoma account for the majority of these effusions, followed by gynecological malignancies and malignant mesothelioma [9]. Our study offers a diagnostic yield of pleural fluid cytology that is a more accurate representation of the patients treated within the United States. Additionally, patient‐specific factors that are unique to this region, such as the prevalence of smoking, access to healthcare, and level of medical noncompliance, will likely result in different proportions of cancer subtypes that cause MPE and hence result in a diagnostic yield per cancer subtype that is different from the existing evidence presented by other countries.

## 2. Materials and Methods

We conducted a retrospective study of all patients with unilateral or bilateral pleural effusions between January 2015 and August 2018. Electronic medical records were obtained through Epic EMR at WakeMed Health and Hospitals. Medical records of patients seen in the adult emergency department and endoscopy unit at WakeMed Health and Hospitals between January 2015 and August 2018 were screened and reviewed in Epic EMR to identify eligible patients. All patients who had undergone thoracentesis and pleural fluid cytological analysis, with or without subsequent pleuroscopy or video‐assisted thoracoscopic surgery (VATS), were included. Data were obtained via retrospective chart review.

We chose patients who had undergone thoracentesis with a confirmed final diagnosis of MPE via cytology or pleural biopsy. We included 667 consecutive patients who underwent pleural fluid sampling with cytologic analysis over a 3‐year period at a tertiary referral center in the United States. Patients with cytology‐negative results who underwent additional diagnostic testing were followed clinically via EMR review for a minimum of 6 months. A “true negative” was defined as the absence of malignant progression or new oncological findings related to the pleural space during this follow‐up period.

We used a standardized Microsoft Excel Enterprise form to collect data on clinical and pleural fluid features. Clinical parameters included age, gender, ethnicity, method of MPE confirmation, smoking exposure, cancer site, and type. Pleural fluid characteristics included volume, pH, lactate dehydrogenase concentration, and polymorphonuclear cell proportion.

### 2.1. Statistical Analysis

**Figure 1:**
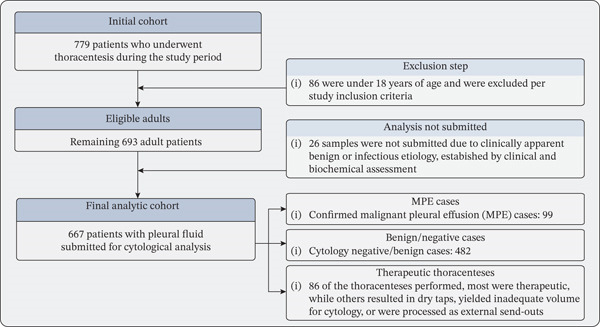
Patient flow diagram. Of the 779 patients who underwent thoracentesis between January 2015 and August 2018, 86 were excluded (age < 18 years). Of the remaining 693 adults, 667 had pleural fluid submitted for cytological analysis. Of these, 99 had confirmed malignant pleural effusion: 86 (86.9%) by cytology, 8 (8.1%) by VATS, and 5 (5.1%) by other methods.

Data were analyzed using SPSS Statistics Version 29.0 (IBM Corp.) and Jamovi Version 2.6.19. Categorical variables were reported as frequencies and proportions. Confidence intervals for sensitivity and specificity were calculated using the Wilson score method. Comparative analyses between cancer subtype groups were performed using chi‐square tests for larger groups (expected cell count ≥ 5) and two‐tailed Fisher′s exact tests for smaller groups. We specifically applied the Yates continuity correction to decrease the risk of Type I error and ensure a more conservative and robust *p* value. A *p* value of < 0.05 was considered statistically significant. Given the retrospective and exploratory nature of this study, no correction for multiple comparisons was applied; results should be interpreted accordingly. The patient selection process is illustrated in Figure 1.

**Figure 2:**
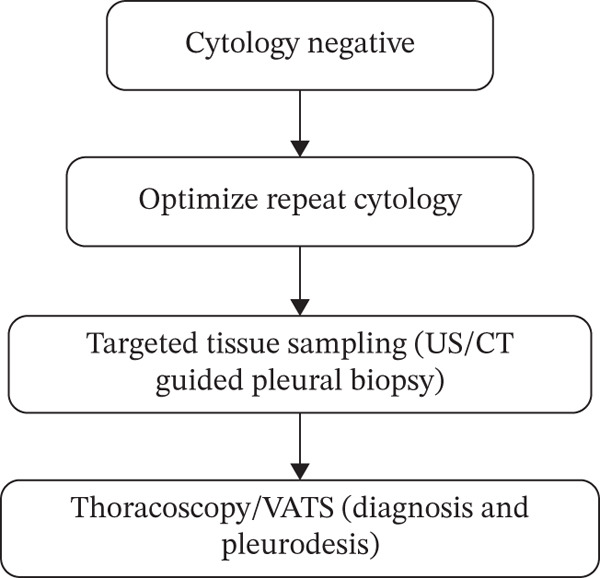
Cytology‐negative diagnostic algorithm: Proposed diagnostic algorithm for the management of cytology‐negative pleural effusion in suspected malignant pleural effusion.

Post hoc Benjamini–Hochberg false discovery rate correction was applied for multiple comparisons

**Figure 3:**
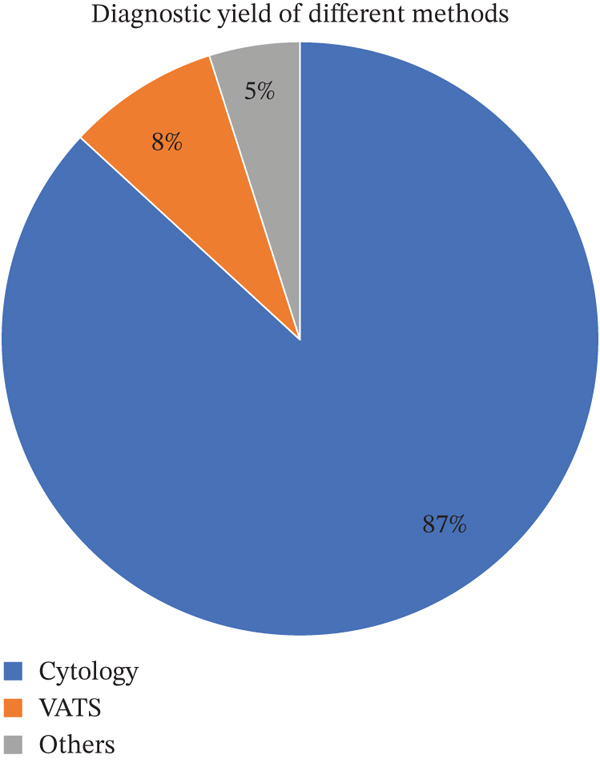
Diagnostic yield of different methods: Diagnostic yield of different investigative modalities in the diagnosis of malignant pleural effusion. Pleural fluid cytology was the primary diagnostic method in the majority of cases (87%), followed by video‐assisted thoracoscopic surgery (VATS) (8%) and other modalities (5%).

## 3. Results

Of the 779 patients who underwent thoracentesis during the study period, 86 were under 18 years of age and were excluded per study inclusion criteria. Of the remaining 693 adult patients, pleural fluid was submitted for cytological analysis in 667 cases; the remaining 26 samples were not submitted due to clinically apparent benign or infectious etiology, established by clinical and biochemical assessment. While none had a documented cancer history. The final analytic cohort comprised 667 patients. Of 667 with cytology: Confirmed MPE cases were 99, cytology negative/benign cases were 482 and 86, respectively. Of the thoracenteses performed, most were therapeutic, whereas others resulted in dry taps, yielded inadequate volume for cytology, or were processed as external send‐outs.

The mean age was 67.8 years (95% CI 66.57–69.17 years), 321 females (50.6%), 313 males (49.4%), and 33 (4.9%) with sex not recorded in the medical record. In our study, 400 were White Caucasian, 142 were African–American (AA), and the rest were Hispanic, Asian, or not Hispanic (NH). Cytology diagnosed 86 (12.89%) of the total cases, whereas VATS diagnosed 8 (1.19%). Out of 99 cases of positive MPE, 86.86% were diagnosed by cytology, 8.08% required VATS, and 5.05% required other investigations such as FNA, IR biopsy, biopsy, EBUS, and peritoneal fluid cytology. In cases where initial pleural fluid analysis is inconclusive, we followed a structured diagnostic escalation, beginning with optimized repeat cytology and moving toward image‐guided biopsy or Video‐Assisted Thoracoscopic Surgery (VATS) if malignancy remained a clinical suspicion (Figure 2). Our data demonstrates that pleural fluid cytology remains the primary diagnostic tool with an 87% yield, while more invasive procedures like VATS or biopsy were required for the remaining 13% of cases (Figure 3).

**Figure 4:**
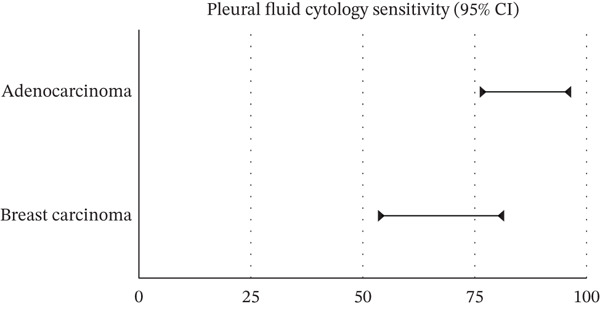
Diagnostic sensitivity of pleural fluid cytology. The graph displays the estimated sensitivity for adenocarcinoma (approximately 76%–96%) and breast carcinoma (approximately 53%–81%).

**Figure 5:**
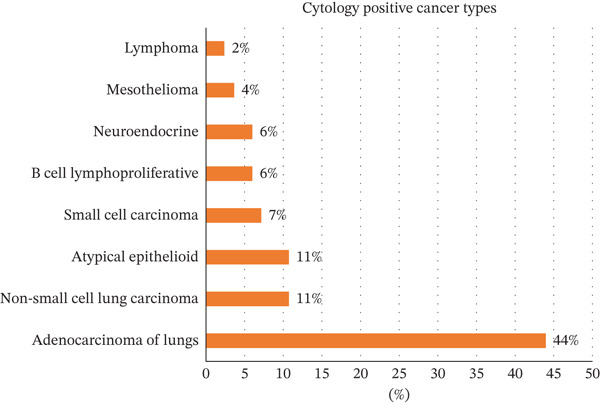
Distribution of specific cancer types among cytology‐positive cases: This figure illustrates the proportional breakdown of cancer types confirmed as cytology‐positive. Adenocarcinoma of the lungs is the most common positive type (44%). Percentages indicate the proportion of each cancer type within the total set of positive cases.

Eighty‐six patients had positive cytology from the first pleural fluid assessment, involving 33 males and 53 females. Sensitivity was highest for adenocarcinoma (90.24%; 95% CI: 83.97%–96.51%). Pleural fluid cytology was most sensitive for diagnosing MPE related to breast cancer (67.44%; 95% CI 53.41%–81.47%) (Figure 4). Among the cases confirmed as cytology‐positive, lung adenocarcinoma was the most prevalent subtype, followed by atypical epithelioid and non‐small cell lung carcinoma (Figure 5.). Among cytology‐positive cases, the most common primary site was breast cancer (*n* = 29; 33.72% of 86), followed by lung cancer (*n* = 14; 16.27%). The overall diagnostic performance of pleural fluid cytology is summarized in Table 1.

**Table 1:** Overall diagnostic performance of pleural fluid cytology for malignant pleural effusion (*N* = 667).

	**MPE confirmed (+)**	**MPE confirmed (−)**	**Total**
Cytology (+)	TP = 86	FP = 1	87
Cytology (−)	FN = 13	TN = 567	580
Total	99	568	667

**Metric**	**Value**	**95% CI**	**Interpretation**
Sensitivity	86.9%	80.5%–91.6%	Detection rate among true MPE
Specificity	99.8%	99.3%–100%	Correctly ruled out among true benign
PPV	98.9%	—	Prob. of true MPE when cytology (+)
NPV	97.8%	—	Prob. of no MPE when cytology (−)

Abbreviations: FN, false negative; FP, false positive; NPV, negative predictive value; PPV, positive predictive value; TN, true negative; TP, true positive.

The overall specificity of pleural fluid cytology for diagnosing MPE was 99.8% (95% CI: 99.3%–100%), with one documented false‐positive case: A patient with empyema whose cytology was reported as malignant glandular cells consistent with adenocarcinoma, subsequently confirmed as nonmalignant on VATS. The positive predictive value (PPV) was 98.9%, and the negative predictive value (NPV) was 97.8%. Diagnostic performance varied substantially by cancer subtype, with adenocarcinoma demonstrating the highest sensitivity (90.2%; 95% CI: 77.5%–96.1%) and skin, prostate, and urothelial carcinomas demonstrating the lowest (13%–14%; Table 2).

**Table 2:** Diagnostic performance by cancer subtype.

Cancer subtype	TP	FN	Sensitivity	95% CI	Clinical note
Adenocarcinoma (lung)	37	4	90.2%	77.5%–96.1%	Highest yield; preferred initial test
Breast cancer (primary)	29	14	67.4%	52.5%–79.5%	Leading primary site in this cohort
Lung cancer (all types)	14	23	37.8%	24.1%–53.9%	Squamous subtypes drive lower yield
Mesothelioma	3	2	60.0%	23.1%–88.2%	Small *n*; VATS/biopsy usually required
Hematological malignancy	5	16	23.8%	10.6%–45.1%	IHC needed; cytology alone insufficient
Skin cancer	2	13	13.3%	3.7%–37.9%	Lowest yield; additional biopsy always required
Prostate cancer	2	12	14.3%	4.0%–39.9%	Frequently cytology‐negative
Urothelial/bladder cancer	1	6	14.3%	2.6%–51.3%	Consistent with literature (~11.8%)

*Note:* Sensitivity of pleural fluid cytology by cancer subtype with 95% Wilson‐score confidence intervals. Subtypes with *n* < 10 should be interpreted with caution given the wide confidence intervals.

Abbreviations: CI, confidence interval; FN, false negative (cytology‐negative, MPE confirmed by VATS/biopsy/other methods); IHC, immunohistochemistry; TP, true positive (cytology‐positive, MPE confirmed by gold standard); VATS, video‐assisted thoracoscopic surgery.

Adenocarcinoma was the most prevalent cytology‐positive histological type, accounting for 43.02% of cytology‐positive MPE cases (*n* = 37 of 86), followed by atypical epithelial cells (9.46%, *n* = 9), B‐cell lymphoproliferative tumors (5.81%, *n* = 5), and neuroendocrine disorders (5.81%, *n* = 5), respectively. Of the 15 cases of lung cancer, nine (60%) were nonsmall cell cancer, one was squamous cell carcinoma, and the remaining six were small cell carcinoma.

**Figure 6:**
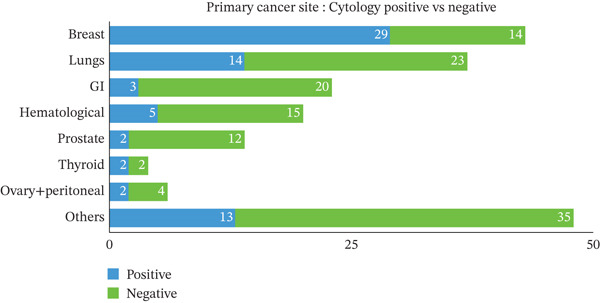
Comparison of primary cancer site: Cytology positive versus negative cases. This stacked bar chart presents the frequency of cytology positive (blue) and negative (green) cases by primary cancer site. Breast cancer (n=29 positive) and lungs (*n*=14 positive) have the highest frequencies of positive cytology, whereas other sites show a lower incidence. Numbers within bars represent raw case counts (*n*).

**Figure 7:**
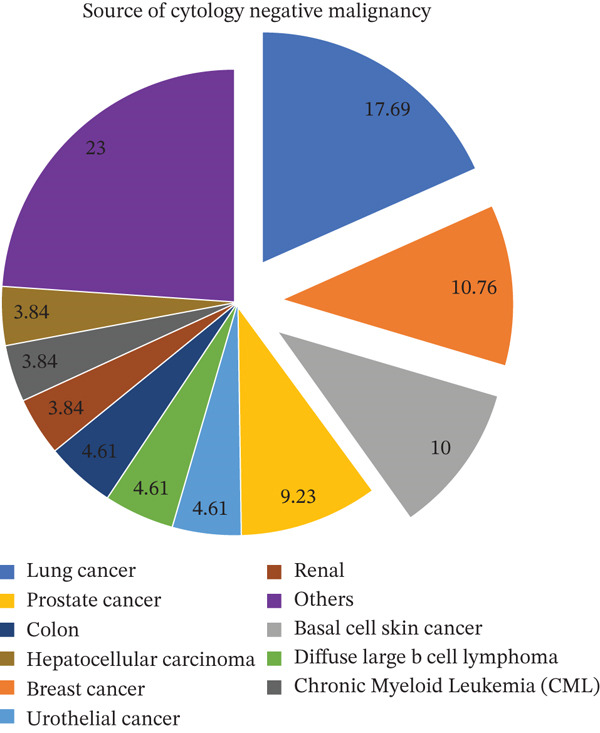
Primary cancer sources for cytology‐negative malignancies. This exploded pie chart shows the breakdown of sources for cases identified as cytology‐negative for malignancy. The percentage values indicate the relative contribution of each primary cancer site to the total group of cytology‐negative samples, with "others" representing the largest aggregated group (23%).

Of the cases in this study, 482 were attributed to benign pleural effusions, with negative cytological findings in all instances. Notably, 130 (27.0% of 482) of these patients had a documented history of cancer, yet pleural fluid cytology remained negative even in the presence of malignancy. The most common primary malignancies among cytology‐negative patients who had cancer were lung cancer (n = 23; 17.69% of 130 cancer patients with negative cytology). Squamous cell lung cancer was the most commonly identified histological type in 10 patients, followed by nonsmall cell adenocarcinoma in nine (only four of them were adenocarcinoma) and small cell lung cancer in three, and two of them were unidentified/lost to follow‐up. Other primary malignancies were 16 (12.30% of 130) hematological malignancies, 14 (10.76% of 130) breast cancers, 13 (10.00% of 130) basal cell skin cancers, and 12 (9.23% of 130) bladder cancers. Ten (7% of 130) were other urothelial cancers. A comparative analysis of primary cancer sites revealed that while lung and breast cancers yielded the highest absolute number of positive results, GI and hematological sites were more frequently associated with cytology‐negative effusions (Figure 6). For the subset of cases that remained cytology‐negative despite confirmed malignancy, the primary sources were most commonly identified as lung and breast origin (Figure 7). Among cytology‐negative patients, we identified 42 patients for additional testing to determine malignancy, but the results were negative. We performed thoracoscopy (with pleural decortication/total decortication/partial decortication/medical thoracoscopy) on 28 patients and VATS (with mechanical pleurodesis/talc pleurodesis) on 6 patients. We also performed medical thoracoscopy (pleuroscopy) on one patient and an IR pleural biopsy on another. Four patients also tested negative for cytology and VATS. In a single thoracentesis, 81 (94.18%) of 86 individuals tested positive for cytology. Six patients had negative cytology in the first procedure but positive in the second and third thoracenteses (4.65% and 1.16%, respectively). On the second cytology, three out of four cases (75%) showed rare atypical cells, which VATS later verified. The histological diagnoses include nonsmall cell adenocarcinoma, metastatic adenocarcinoma of pulmonary origin, and myxoid adenocarcinoma, indicating that 75% of the cases were classified as adenocarcinomas. Another single case was malignant mesothelioma. Malignant mesothelioma was also found in a single case detected during the third cytology and then confirmed by VATS. We performed VATS on 9 of the 86 cytology‐positive cases to histologically confirm or subclassify the malignancy.

We performed VATS in three cases after initial cytology revealed a positive result: They included biphasic mesothelioma, metastatic adenocarcinoma, and metastatic adenocarcinoma of breast cancer. We performed VATS following second cytology in five patients: malignant mesothelioma, adenocarcinoma of breast origin (two cases), chondrosarcoma, and metastatic adenocarcinoma of pulmonary origin. In a single case, we performed VATS following the third cytology, which revealed malignant mesothelioma.

In this study, of the 36 cytology‐negative patients who underwent VATS, eight (22.22%) had positive findings. This subgroup had a mean age of 68.6 years, with equal sex distribution (four male and four female). The group included four patients with adenocarcinoma (50%), one with small cell carcinoma, and others with epithelioid and nonsmall cell lung cancer. These patients presented various primary sites of malignancy, including two cases from the breast and one from the kidney. Five of the cases in our study were recognized by FNA, interventional radiography (IR), biopsy, EBUS, and peritoneal fluid cytology, but they were not identified by cytology or VATS.

We identified a single case of empyema, where cytology revealed malignant glandular cells consistent with adenocarcinoma of the lungs, but VATS results were negative. VATS revealed membranous fibropurulent debris consistent with empyema, and no evidence of malignancy. Baseline clinical and demographic characteristics of the full study cohort, including detailed cytology‐positive and cytology‐negative cancer subtype distributions, are presented in Tables 3–6.

**Table 3:** Baseline characteristics and pleural fluid cytology results of the study cohort (*N* = 667).

Variable	Frequency (%)
Age, mean (years)	67.87
Sex: female	321 (50.6)
Ethnicity	
White–C	400 (59.79)
AA	142 (21.22)
Hispanic	19 (2.84)
NH	18 (2.69)
Asian	7 (1.04)
American Indian	2 (0.29)
Middle Eastern	1 (0.06)
Method of confirming malignant effusion	
Cytology	86 (86.86%)
VATS	8 (8.08%)
Others	
‐FNA	5 (5.05%)
‐IR biopsy	
‐Biopsy	
‐EBUS	
‐Peritoneal fluid cytology	

*Note:* Values are presented as *n* (%) unless otherwise stated.

Abbreviations: AA, African–American; EBUS, endobronchial ultrasound; FNA, fine needle aspiration; NH, non‐Hispanic; VATS, video‐assisted thoracoscopic surgery.

**Table 4:** Demographic and clinical characteristics of patients with cytology‐positive malignant pleural effusions.

**Cytology positive**	**86**
Female: male	53:33 (1.6:1)
White—c	39 (45.34%)
AA	33 (38.37%)
Others	14 (16.27%)
Smoking history	
Current	17 (19.76%)
Former	35 (40.69%)
Never	34 (39.53%)
Effusion	
Exudative	71(77.2%)
Transudative	7 (7.6%)
Unknown	8 (9.3%)

**Confirmed on which cytology**	
First	81 (94.18%)
Second	4 (4.65%)
●Nonsmall cell adenocarcinoma	●1
●Rare atypical epithelial cells (metastatic adenocarcinoma of pulmo origin/myxoid chondrosarcoma/malignant mesothelioma)	●3
Third	1 (1.16%)
●Rare atypical cells (malignant mesothelioma)	

**Cytology-positive cancer types:**	
Adenocarcinoma of lungs	37 (43.02)
Nonsmall cell lung carcinoma	9 (10.46)
‐squamous cell carcinoma	1 (11.11, of NSCLC)
Small cell carcinoma	6 (6.9)
Atypical epithelioid	9 (10.46)
B cell lymphoproliferative	5 (5.81)
Neuroendocrine	5 (5.81)
Mesothelioma	3 (3.48)
Lymphoma	2 (2.32)
Others(plasma cell, ductal adenocarcinoma, urothelial, chronic lymphocytic leukemia(CLL), chondrosarcoma, mantle cell, multiple myeloma, abundant small lymphocytes)	10 (11.62)

**Primary source of cancer**	
●Breast	●29 (33.72)
●Lungs	●14 (16.27)
●Hematological malignancy (CLL, Hodgkin, non‐Hodgkin′s)	●5 (5.61)
●GI	●3 (3.48)
●Prostate	●2 (2.32)
●Thyroid	●2 (2.32)
●Ovarian/tubal/peritoneal	●2 (2.32)
●Others	●13 (15.11)

*Note:* Others include skin, renal, nose, cervical, and bone.

Comparative analysis revealed that breast cancer as a primary site was significantly more likely to yield a cytology‐positive result than lung cancer (all types combined; chi‐square with Yates′ correction: *χ* ^2^ = 5.87, df = 1, *p* = 0.015). Hematological malignancies demonstrated significantly lower cytology sensitivity compared with all other cancer types combined (Fisher′s exact test: OR = 0.015, 95% CI: 0.004–0.061, *p* < 0.001). No statistically significant difference in sensitivity was observed between mesothelioma and adenocarcinoma (Fisher′s exact: OR = 0.16, *p* = 0.120), though this comparison was limited by the small number of mesothelioma cases (*n* = 5). A summary of all comparative statistical analyses is presented in Table 7. Post hoc Benjamini–Hochberg false discovery rate correction was applied to the same table for multiple comparisons.

**Table 5:** Primary malignancy sites and histopathological subtypes in patients with cytology‐negative pleural effusions.

Cytology negative	
**Lung cancer**	**23 (17.69)**
●Squamous cell carcinoma	●10 (43.47)
●Nonsmall cell lung cancer	●5 (21.73)
●Adenocarcinoma	●4 (17.39)
●Small cell lung cancer	●2 (8.69)
●Lung cancer (unknown/lost to f/u)	●2 (8.69)
Breast cancer	14 (10.76)
Basal cell skin cancer	13 (10.00)
Prostate cancer	12 (9.23)
Urothelial cancer	6 (4.61)
Diffuse large B cell lymphoma	6 (4.61)
Colon	6 (4.61)
Renal	5 (3.84)
Chronic myeloid leukemia (CML)	5 (3.84)
Hepatocellular carcinoma	5 (3.84)
Rectal	3 (2.30)
Pancreatic adenocarcinoma	2 (1.53)
Stomach adenocarcinoma	2 (1.53)
Carcinoid tumor	2 (1.53)
Endometrioid clear cell cancer	2 (1.53)
Epithelioid mesothelioma	2 (1.53)
Papillary thyroid carcinoma	2 (1.53)
Malignant melanoma	2 (1.53)
Hodgkin lymphoma	2 (1.53)
Others	11 (8.46)

*Note:* Others include gastric lymphoma, ovarian cancer, myeloproliferative, Acute Lymphocytic Leukemia (ALL), Acute myeloid leukemia (AML), CLL, esophagus/parotid/tongue squamous cell carcinoma, adrenal adenoma, leiomyoma of the uterus.

**Table 6:** Secondary diagnostic procedures and histopathological outcomes in cytology‐negative cases.

Cytology negative cases that are suspected of malignancies are further tested by the following procedure.	
**VATS (with mechanical/talc pleurodesis)**	**8**
Thoracoscopy (with total/partial decortication)	28
Pleuroscopy	1
IR biopsy	1
**Cytology negative, VATS positive**	
Sex ratio	1:1
White–C	5 (62.5%)
AA	3 (37.5%)
Smoking history	
●Current	2 (25%)
●Former	4 (50%)
●Never	2 (25%)
Adenocarcinoma	4 (50.00)
Nonsmall cell lung cancer	1 (12.50)
Epithelioid tumor	1 (12.50)
Small cell cancer	1 (12.50)
**Primary site:**	
●Breast	●2 (25.00)
●Renal	●1 (12.50)
**Cytology and VATS negative, other finding positive**	
FNA	1
IR Biopsy	1
Biopsy (primary site: prostate)	1
EBUS (primary site: lung)	1
Peritoneal fluid cytology	1

**Table 7:** Summary of comparative statistical analyses between cancer subtype groups.

Comparison	Data used	Test	Statistic	*p*value	BH‐adjusted *p* value	Interpretation
Breast versus lung cytology yield	2 × 2 contingency [29,14; 14,23]	Chi‐square (Yates)	*χ* ^2^ = 5.87, df = 1	*p* = 0.015	*p* = 0.030	Breast cancer is significantly more likely to yield cytology‐positive MPE than lung cancer (all types); remains significant after FDR correction
Hematological versus all other malignancies	2 × 2 contingency [5,16; 81,4]	Fisher′s exact	OR = 0.015	*p* < 0.001	*p* = 0.004	Hematological malignancies have markedly lower cytology sensitivity versus all other cancer types; remains highly significant after FDR correction
Mesothelioma versus adenocarcinoma sensitivity	2 × 2 contingency [3,2; 37,4]	Fisher′s exact	OR = 0.16	*p* = 0.120	*p* = 0.160	No significant difference (small *n*) even after FDR correction; VATS remains recommended for mesothelioma regardless
VATS yield (cytology‐neg patients)	8/36 positive	Descriptive	22.2%	—		In cytology negative patients with clinical suspicion, VATS adds diagnosis in approximately one in five cases
Repeat cytology (second thoracentesis)	6/86 positive on second	Descriptive	7.0%	—		Repeat thoracentesis resolves 7% of initial false‐negatives; consistent with Arnold et al. (5.6%)

*Note:* Chi‐square test with Yates continuity correction was used for the breast versus lung comparison (expected cell counts ≥ 5); two‐tailed Fisher′s exact test was used for smaller groups. Benjamini–Hochberg false discovery rate correction (Q = 0.05) was applied to all three hypothesis tests. BH‐adjusted *p* values are reported alongside original *p* values. Descriptive analyses (VATS yield and repeat cytology yield) were not subject to correction.

Abbreviations: BH, Benjamini–Hochberg; df, degrees of freedom; FDR, false discovery rate; OR, odds ratio.

## 4. Discussion

This study analyzes the prevalence and sensitivity of pleural fluid cytology in patients with pleural effusion, which later confirmed various primary and metastatic malignancies. We performed VATS, FNA, pleural biopsy, and EBUS to confirm the presence of malignancy in cytology‐negative patients in whom malignancy was suspected based on their history and clinical suspicion. We also performed these tests on cytology‐positive patients to confirm the presence of malignancy to rule out false positive results. A key finding of our investigation was that tumor cell type affected pleural fluid cytology diagnostic accuracy.

In general, pleural fluid cytology successfully identified 86.9% of all confirmed MPE cases in this cohort. Of these, adenocarcinoma of the lung had the highest sensitivity (90.24%), followed by nonsmall cell lung cancer and atypical epithelial cell cancer subtypes originating from breast cancer, which showed the highest prevalence of cytology‐positive cases followed by lung cancer and hematological malignancies. A study conducted by Porcel et al. on 3000 thoracentesis samples showed that most of the initial tumors that spread to the pleural space were lung and breast cancer together [10]. Moreover, many studies have indicated that the lungs are the most common primary site responsible for MPE. However, in our study, breast cancer (33.7%) emerged as the leading primary cancer site responsible for positive cytology in MPE, with rates nearly double those of lung cancer (16.27%). The known prolonged survival of breast cancer increases the cumulative probability of developing pleural metastases, potentially explaining the higher representation in our cohort compared with older historical series. This demographic skew is further evidenced by our 1.6:1 female‐to‐male ratio among cytology‐positive patients.

Furthermore, compared with previous research from the last 22 years, where the sensitivity of cytology ranged from 39% to 79%, ours was higher. Furthermore, those studies indicate that mesothelioma exhibited reduced sensitivity to cytology; however, in our study, lymphoma showed even lower sensitivity to cytology compared with mesothelioma [3, 11]. The conventional methods for confirming a diagnosis of hematological malignancies through pleural fluid cytology and biopsy are quite challenging, and these methods often have a low diagnostic yield [12]. Our study identified 5.81% of hematological malignancies from pleural effusions, with MPEs positively contributing to cytology. Out of 18 hematological malignancies, 5 cases tested positive for cytology, resulting in a sensitivity of 23.8% (5 of 21 confirmed hematological MPE cases) for cytology‐based diagnosis. Combining cytology with immunohistochemistry increased the sensitivity of diagnosing hematologic malignancies by 59.1% [13]. In contrast to adenocarcinoma, hematological malignancies exhibited markedly lower cytology sensitivity (23.8%; 95% CI: 10.6%–45.1%), significantly lower than all other cancer types combined (Fisher′s exact test, OR = 0.015, *p* < 0.001). This underscores the need for immunohistochemistry supplementation when hematological malignancy is suspected.

The most common occurring subtype of lung cancer, squamous cell carcinoma, frequently shows negative cytology. Furthermore, cancers arising from the skin, prostate, and breast require additional diagnostic tests. In cases where the pleural cytology from thoracentesis was negative, we considered a pleural biopsy using medical pleuroscopy or VATS. In squamous cell carcinoma with malignant effusion, pleural fluid cytology exhibited a poor diagnostic yield. These results are consistent with those reported in the literature [14].

In a prior study, out of 1172 lung cancer cases, 11 individuals (0.94%) had cutaneous metastases confirmed by skin biopsy [15]. In our study, we found that 15 cases (15.15%) out of 99 were diagnosed with skin as a primary site of MPE, which suggests a rising trend of skin cancer manifesting as a pleural effusion by pulmonary metastasis; among them, 13 were cytology negative, that is, approximately 87% of total skin cancer cases. This warrants further investigations in cases where pleural effusion is caused by basal cell carcinoma.

The cytological yield (sensitivity) of MPE for urothelial carcinoma is around 11.8%, indicating a comparatively low rate [11]. In our study, prostate and urothelial cancer contributed 9.23%, whereas cytology results were negative in 4.61% of cases. This indicates that the cytological yield is also lower in our cases. Cytology serves as a crucial and effective preliminary assessment for the diagnosis of MPE. Research by Arnold et al. has shown that repeated fluid cytology in nondiagnostic malignant cases results in a diagnosis for 5.6% of MPEs [16]. We also concluded that the diagnostic efficacy increases with successive thoracenteses, and the tumor type significantly impacts the cytological diagnosis.

In a cohort of MPE ranging from different etiologies, Porcel et al. found an overall sensitivity of pleural fluid cytology obtained through thoracentesis to be 51%, with an increase to 59% on the next thoracentesis [10]. Similar studies conducted by Prakash et al. revealed that pleural fluid cytology achieves a sensitivity of 60% in diagnosing lung cancer following the initial thoracentesis. Repeatedly, this value rose above 75% [17]. However, our study showed 81.81% (81/99) with an increase to 86.86% (86/99) on subsequent thoracentesis.

We performed VATS in 36 patients with cytology‐negative pleural effusions, among which 8 (22.22%) cases were positive. While thoracoscopy had a diagnostic sensitivity of 95% for cancer in previous studies by Harris et al. [18], in our cases, its sensitivity was 61.53%. We performed other tests where cytology and VATS both were negative. FNA, IR biopsy, EBUS, and peritoneal fluid cytology contribute vitally to diagnosing MPE in both cytology‐ and VATS‐negative cases. In patients with MPE, needle biopsy of the pleura yields a diagnosis between 39% and 75% [19]. During our investigation, we successfully detected three cytology‐negative patients with negative VATS using various biopsy techniques, including IR and FNA, with a sensitivity of 60%. Advanced gynecological malignancies frequently manifest as malignant ascites (MA), with a significant number of these individuals also developing MPEs [20]. In one of our cases of peritoneal carcinomatosis, neoplastic cells were not detected through pleural cytology, thoracoscopy of pleural fluid, or tissue biopsy, and finally a peritoneal biopsy was needed.

## 5. Limitation

This study has several limitations that should be considered when interpreting these findings. The retrospective single‐center design at a tertiary referral hospital introduces potential selection bias; patients referred to this center may have more advanced or diagnostically challenging malignancies, which could inflate the observed cytology sensitivity compared to community practice. Cytology specimens were reviewed without a formal interrater reliability assessment, and variability in pathologist interpretation cannot be excluded. The study lacks long‐term follow‐up data; survival outcomes and clinical response to treatment were not captured, limiting the prognostic implications of the findings. Patients with cytology‐negative results and low clinical suspicion for malignancy did not undergo VATS, which may have led to underestimation of the true false‐negative rate. Additionally, 26 patients whose pleural fluid was not submitted for cytological analysis were classified as benign based on clinical and biochemical assessment alone; although none had a documented cancer history, occult malignancy cannot be entirely excluded in this subgroup. Despite these limitations, this study provides valuable real‐world US data on cytology diagnostic yield across cancer subtypes in a large contemporary cohort.

## 6. Conclusion

Overall, our study demonstrated higher cytology sensitivity (86.9%) than that reported in existing literature from recent years for diagnosing MPE, and our results demonstrate that the diagnostic yield of pleural fluid cytology, VATS, and biopsy is significantly impacted by the underlying tumor type. Cytology is highly sensitive for MPE caused by adenocarcinoma (90.2%), particularly lung adenocarcinoma, and shows moderate sensitivity for breast cancer (67.4%). Sensitivity is poor for hematological malignancies (23.8%), lymphoma, skin cancer, prostate cancer, and urothelial carcinoma (< 15%), where supplementary techniques such as immunohistochemistry, EBUS, or VATS are essential.

**Nomenclature**
AA: African–American
ALL: acute lymphocytic leukemia
AML: acute myeloid leukemia
BH: Benjamini–Hochberg
CLL: chronic lymphocytic leukemia
CML: chronic myeloid leukemia
df: degrees of freedom
EBUS: endobronchial ultrasound
EMR: electronic medical record
FNA: fine‐needle aspiration
FP: false positive
IHC: immunohistochemistry
IR biopsy: interventional radiology biopsy
MPE: malignant pleural effusion
NH: non‐hispanic
SPSS: statistical package for social sciences
VATS: video‐assisted thoracoscopic surgery
BH: Benjamini–Hochberg

## Author Contributions

Santosh Basyal: conceptualization, methodology, data curation, writing—original draft. Antony D. Rawindraraj: data curation, formal analysis. Rahul Parajuli: writing, review, supervision, and editing, final draft. Shardul Bhattarai: writing—review & editing. Laxman Wagle: data curation, writing—review & editing. Kritick Bhandari: formal analysis, writing—review & editing. Vikas Pathak: methodology, writing—review & editing.

## Funding

No funding was received for this manuscript.

## Disclosure

All authors have read and agreed to the published version of the manuscript.

## Ethics Statement

This study was conducted in accordance with the Declaration of Helsinki and was approved by the WakeMed Health and Hospitals Institutional Review Board. Given the retrospective nature of the study and the use of de‐identified electronic medical records, the requirement for individual informed consent was waived by the IRB.

## Conflicts of Interest

The authors declare no conflicts of interest.

## Data Availability

The data that support the findings of this study are available from the corresponding author upon reasonable request.
